# Magnitude of emergence agitation, its interventions and associated factors among paediatric surgical patients

**DOI:** 10.1186/s12871-024-02623-5

**Published:** 2024-07-13

**Authors:** Habtamu Tilahun Aniley, Samrawit Tassew Mekuria, Mebratu Abraha Kebede, Alembrhan Hagos Gebreanania, Mahteme Bekele Muleta, Tafere Tilahun Aniley

**Affiliations:** 1 Department of Anesthesia, MeQrez General Hospital, Addis Ababa, Ethiopia; 2 Department of Anesthesia, St. Paul’s Hospital Millennium Medical College, Addis Ababa, Ethiopia; 3 Research Directorate office and Nursing Education Department, Saint Paul’s Hospital Millennium Medical College, Addis Ababa, Ethiopia; 4 Department of Anesthesia, Mekele University, Addis Ababa, Ethiopia; 5 Department of Statistics, University of South Africa, c/o Christiaan de Wet Road & Pioneer Avenue, Johannesburg, South Africa

**Keywords:** General anaesthesia, Emergence agitation, Excitation, Magnitude, Paediatrics, Pain

## Abstract

**Background:**

Emergence agitation is a transient confusional state of a child associated with consciousness from general anaesthesia, commonly occurs in the postoperative setting which delays their recovery and exposes them to traumas. The main objective of the current study was to investigate the magnitude of emergence agitation, its interventions and associated factors among paediatric surgical patients at Saint Paul’s Hospital Millennium Medical College, Addis Ababa, Ethiopia.

**Methods:**

Hospital based cross-sectional study with prospective follow-up framework was conducted on a paediatric surgical patients aged 2-14 years who underwent surgery under general anaesthesia between June 1 - October 30 2022. Stratified sampling method followed by simple random sampling technique was employed to reach study participants. Magnitude of emergence agitation and its interventions done at post-anaesthetic care units were recorded. Data analysis was carried out using a descriptive statistics method and the results were summarized using tables and diagrams. Bivariate analysis was done to identify causal relationship and multivariable analysis to assess the confounding effects of factors associated with emergence agitation. A *p*-value of less than 0.05 was considered statistically significant factor.

**Results:**

A total of 150 participants were included in the current study, where 107 (71.3%) were male and 97 (64.7%) were preschool aged. About 81 (54%) of care givers were female and majority of them have completed primary school. The mean (standard deviation) age of the participants was 6.4 (3.57) years. Around 42.7% of them developed emergence agitation with an average duration of 8.39 ± 4.45 minutes. Factors such as propofol administration at the end of procedure (OR of 0.104 with 95% CI [0.035, 0305]), Ear, nose, throat surgery and oral maxillofacial surgery (OR of 2.341 with 95% CI [1.051, 5.211]) and arrival of patient to recovery awake (OR of 0.456 95% CI [0.209, 0.994]) showed statistically significant association with emergence agitation.

**Conclusion:**

Almost half of the study participants experienced emergence agitation which is high magnitude. Ear, nose, throat surgery and oral maxillofacial surgeries were predictive factors of emergence agitation while propofol administration at the end of procedure and arrival of patient to recovery awake significantly decreased risk of emergence agitation. Therefore, anaesthesia personnel should have essential skills and knowledge to effectively care for children perioperatively including to minimize and treat emergence agitation.

## Background

The concept of Emergence Agitation (EA) also known as Emergence Excitement was described in the late 1960s by Eckenhoff referring to the transient confusional state of a child associated with consciousness from general anaesthesia and surgery [1]. In the recent studies, EA have been generally described as mental health complications observed during recovery from anaesthesia [2]. In the literature, the concepts of EA and delirium have been used interchangeably, however there is a difference in terms of their definition and practices. Emergence delirium (ED) is complex psychiatric syndrome, while EA is a state of mild restlessness and mental distress [2, 3]. Unlike delirium, EA typically resolves quickly and is followed by uneventful recovery. It usually occurs within the first 30 minutes of recovery but can have late onset in children who are brought to the recovery room asleep and might lead to serious health complications if not treated. However, many children who arrive to post-anaesthetic care unit (PACU) asleep experience agitation later during recovery [4].

Paediatric patients after surgical operation with general anaesthesia commonly develop EA in the postoperative period. Thus, agitated patients require close follow-up, focused history, and physical examination to evaluate the development of agitation and provide management tools to control the patient’s agitation [3]. Repeated assessments of behaviour following anaesthetic recovery of patients were the requirements to define an incidence and duration of EA.

Preoperative anxiety and EA have been associated with an increased risk of transient postoperative maladaptive behaviours (PMB). Maladaptive behaviours exhibited by children can range from increased general anxiety to sleep disturbances, night-time crying, separation anxiety, temper tantrums, and enuresis. However, the relationship between the presence of EA and PMB is complex and influenced by confounding factors such as preoperative anxiety level in both the patient and the parent [5].

Paediatric patients agitate with different types and forms of symptoms after going through surgery with general anaesthesia. Despite most paediatric patients undergoing minor surgical procedures after general anaesthesia, recent reports show higher incidence rate of EA, some of them even showing no sign of pain or stimulus. This might result clinically significant consequences to the patient’s, such as injury to themselves or to the caregivers, falling, accidental removal of drains or intravenous catheters or invasive monitors, and depression. In addition, it might also increase the workload of the anaesthesiologist, PACU nurses, parents or attendants and incur high medical care costs [6]. Further, EA also increases the recovery time of the paediatric patients in the post-anaesthesia recovery room. Furthermore, paediatric patients with EA might have a longer time to stay in the hospital which has its own economic burden to the parents or caregiver and the country [6].

Further, the optimal strategy for management of EA should focus primarily on prevention and mitigations of modifiable risk factors. Non-pharmacologic interventions have been shown to be effective for alleviating preoperative anxiety and reducing postoperative agitation. Preoperative education of children and their parents, distraction with age-appropriate interventions (toys, games, videos, clowns), music therapy and hypnosis are all potentially beneficial. In addition, clinicians should emphasize treatment of untoward events such as nausea, emesis, hypothermia, and shivering [7]. The pharmacologic management options include sedative drugs, opioids and NSAIDS were known to decrease the adverse events associated with EA.

Few studies investigated the associated risk factors and management of EA in Ethiopia [8, 9]. Ethiopia is one of the country with high EA incidence rate and it is reported to poses a challenge specifically for nurses and PACU provides. A study in Gonder Town, Ethiopia, shows that the incidence rate was more than fifty percent [8]. A similar study done at Addis Ababa University on four governmental hospitals, the incidence rate of EA was around 49.0%. Most of the patients have developed agitation within 15 minutes and 21% of agitated patients experienced self-harm [9]. Sociodemographic characteristics such as age (2-5 years) and gender were identified potential risk factors of EC in children [8, 10, 11]. Furthermore, previous surgery status, preoperative anxiety, parental anxiety, child and parent interaction with healthcare providers are among the commonest patient related factors affecting EA [11]. This is a common problem an anesthetists, nurses, or physicians encounter immediately after anaesthesia and in the PACU in pediatric patients. Thus, it is worth to investigate the magnitude of EA for pediatric patients undergoing surgery under general anaesthesia, and what factors are associated with postsurgical children’s EA.

Due limited data availability on emergence agitation, most studies concentrate on the development of agitation following discharge from the PACU to either intensive care unit (ICU) or outpatient care. However, few studies highlight the extent of EA in the general surgical population at the emergence phase, which occurs immediately after admission to the PACU. Therefore, this study is intended to investigate the associated factors of EA and assess the intervention strategies applied for managing the complications developed due to EA for paediatric patients undergoing surgical operation with general anaesthesia at Saint Paul’s Hospital Millennium Medical College, Addis Ababa, Ethiopia.

## Methods

### Study area

The study was conducted at Saint Paul’s Hospital Millennium Medical College (SPHMMC) located in Addis Ababa, the capital city of Ethiopia. The college is currently serving as a teaching hospital and contains eighteen operation theatres, i.e., six from major OR, four obstetric OR, two ENT and maxillofacial OR, two ophthalmologic OR, and four AaBET hospital. AaBET hospital is a branch of SPHMMC which serves for orthopedic, plastic and neurosurgical patients. There are total of 5 post-anesthesia recovery rooms at its respective operation theater.

### Study design and period

A hospital based cross-sectional study design with prospective follow-up framework was applied to assess the magnitude of emergence agitation, its intervention and associated factors among pediatric surgical patients undergoing surgery under general anesthesia at SPHMMC. The study was conducted from June 1, 2022 to October 30, 2022. Over the last six months, a total of 470 paediatric patients underwent surgery under general anesthesia at SPHMMC. Of these, 180 were from pediatric surgery, 187 were from ENT and maxillofacial surgery, 54 were from ophthalmologic surgery department, and 49 were from AaBET hospital.

### Sample size and sampling technique

The sample size was calculated with single population proportion using $n=\frac{(Z_{(\alpha /2)})^2 P(1-P)}{\epsilon ^2} = 196$ where *p* is the population proportion of EA which is 52.3%, $\epsilon$ is degree of accuracy 7%, and $Z_{(\alpha /2)}$ is the 95% level of confidence [8, 12]. Since, the ratio of sample size to population size is larger than 5%, the sample size was adjusted $n = \frac{196}{1+\frac{196}{470}} = 138$. Adding 10% non-response rate, the final sample size was set to 150. Using stratified sampling to each department and proportional allocation techniquea, from pediatric surgery 57, from ENT and OMF surgery 60, from ophthalmology surgery 17 and 16 of them were from plastic, orthopedic, neurosurgery department at Aabet hospital. Simple random sampling technique (lottery method) was employed to reach study participants from operation theatre’s location and department.

### Inclusion and exclusion criteria

#### Inclusion criteria

All paediatric patients who were classified as ASA 1 and ASA 2 age between 2-14 years old, and who underwent surgery under general anaesthesia during study period were included in the current study.

#### Exclusion criteria

Children with febrile illness before surgery, directly transferred to paediatric intensive care unit (PICU), and ASA classification of 3 or more were excluded.

### Response and predictors

The response variable considered in this study was the binary outcomes of emergence agitation status categorized as “yes” or “no” if the child develops or does not develop emergence agitation, respectively. The PAEDS scale which incorporates cognitive and agitation assessment items, is generally acknowledged to be the most valid and reliable tool to evaluate EA [13, 14]. It evaluates various behavioral dimensions including (i) the child’s ability to make makes eye contact with care givers, (ii) child actions is purposeful, (iii) child is aware of his/her surrounding, (iv) child is restless, and (v) child is inconsolable. The trained PACU nurse rate the patient in each category using “Not at all” , “Just little”, “Quite bit”, “Very much” , and “Excellent” assessments. The first three items are rated on a scale of 4 to 0, while the last two items are rate from 0 to 4. A score of 4 indicates the most favorable behavior, while a score of 0 indicates the least desirable behavior. The sores are aggregated to derive the total PAED score ranging from 0 to 16. A PAEDS score exceeding 12 indicates that the patient has developed EA and the response was classified as “yes” or “no”. Then, the response coded the values “1” for a “yes” or “0” for a “no” responses for further analysis.

The predictor variables considered were (i) Socio-demographic characteristics related to parents or caregiver such as gender (male, female), educational status (not educated, primary school, secondary school and college/university), employment status (not employed, employed) and age (years), (ii) Socio-demographic characteristics related to the paediatric patient such as age (in years), gender (male, female), weight (kg), (iii) Clinical and management related variables such as ASA classification(ASA 1 , ASA 2), premedication (yes, no), type of surgery (general pediatric surgery, ENT and OMF surgery, plastic surgery, orthopedic surgery, neurosurgery, opthalmologic surgery), duration of surgery (in minutes), techniques of GA such as GA with facemask (GA/Facemask), GA with laryngeal maskairway (GA/LMA), and GA with an endotracheal tube (GA/ETT), anaesthesia induction agent (propofol, ketamine, ketofol, thiopental, halothane, other), analgesia techniques (opoids, non-opoids, caudal), extubation techniques (awake extubation, deep extubation), anaesthesia time (in minutes), propofol administration after closing inhalational agents at the end of surgery (yes, no), EA durations (in minutes), time stayed in the PACU (in minutes), (iv) Psychological related variables such as difficult separation behaviour (good separation, difficult separation), and (v) Management related variables such as parent entry to PACU (yes, no), restrains (yes, no), nonpharmacologic treatment (yes, no), and pharmacologic treatment (yes, no). Patient age was categorized as $2-7$ years and $\ge 7$ years to classify child in to preschool or school age, respectively. Difficult parental separation behaviour was assessed observing child’s who was asleep, good separation, awake crying and easily reassured, and crying cannot be reassured was scored from 1 to 4, respectively. A child who had 3 and 4 scores was considered a difficult separation. Measurement of difficult parental separation behaviour will be either difficult separation or good separation. Further, the functional status of child was classified based on the reference guidelines of the American Society of Anaesthesiology (ASA) [15].

### Data collection procedure

To ensure quality of data, data collection procedure was carried out using English version structured questionnaires. Pre-test was done using 5% of sample size. Based on the findings of the pre-test, amendments were incorporated on the questionnaire to avoid unnecessary confusion, errors, and inconsistencies to maintain the quality of the data. The final questionnaire was translated to local languages such as Amharic and Afan Oromo to collect data from native speaker parents or caregivers. A total of four year I and year II Anaesthesiology, Critical Care residents and four PACU nurses were recruited to collect data. The data collectors were trained on the aim of the study, mechanisms to approach study participants, and the use of questionnaire to collect data. Consent for the study and sociodemographic characteristics of the parent or caregiver were collected preoperatively by giving a written questionnaire to the parent or caregiver which was also translated in Afan Oromo and Amharic language. Other preoperative data were collected from the patient’s preoperative evaluation paper attached to the patient chart and observation by residents before the patient was taken to the operation theatre. In addition, the trained residents also collect intraoperative patient data from anaesthesia follow-up sheet. Postoperative, patients were monitored in the PACU for the first 30 minutes by the trained PACU nurses. They measured the PAEDS score every 10 minutes, and trained residents supervised the data collection process. Furthermore, any intervention done for those patients who developed EA were recorded. The principal investigator supervised the data collection procedure for completeness, accuracy, and clarity. Finally, data was cleaned, coded, entered SPSS Version 22 and cross checked before the actual data analysis.

### Data analysis

The collected data was coded, cleared and stored using SPSS Version 22 for doing statistical analysis. Descriptive statistics was performed to determine the frequency and percentage distribution of the character, and the results were presented in the form of tables, graphs, and diagrams. Further, an inferential statistics method was carried out to identify associated risk factors by fitting a binary logistic regression model to calculate the crude odd ratio (COD) and a multivariable logistic regression model to calculate the adjusted odd ratio (AOR) [12]. Pearson chi-square test statistics and associated *p*-value were done for each variable and a *p*-value less than 0.25 were included to conduct the multivariable analysis [12]. Finally, both the COR and AOR with their corresponding 95% confidence interval for the estimate of the parameter of interest were reported. If the confidence interval of both COR and AOR includes the value 1, it was reported that the factor does not have an effect of emergence agitation of children, otherwise it’s considered to be a significant factor associated with emergence agitation.

### Operational definitions

 

1. Unless specified, the term “child” refers to a person between the ages of 2 to 14 years old.
2. Difficult parental separation behaviour: It is the child’s behaviour while the operation room staff trying to take the child to the operation theatre.
3. State of arrival is the level of consciousness and intubation status on arrival to PACU either intubated and asleep, extubated and asleep, or extubated and awake.
4. Duration of anaesthesia indicates the time between induction of anaesthesia to transfer the patient to PACU.
5. Duration of surgery indicates time between incision to dressing of the surgical wound.
6. General anaesthesia defines the state produced when a patient receives medications to produce amnesia and analgesia with or without reversible muscle paralysis.

## Results

### Sociodemographic characteristics of participants

A total of 150 paediatric surgical patients who underwent surgery under general anesthesia were included in the study, of which 107 (71.3%) were male, and 97 (64.7%) were preschool age patients. The age of pediatric surgical patients included in the study ranges from 2 to 14 years old with mean (standard deviation) of 6.4 (3.57) years. The weight of paediatric patients range between 8 kg and 50 kg with mean (SD) of 20.3 (7.59) kg (Table 1). The demographic characteristics of parents or caregivers of paediatric patients were also presented in Table 1. The result show that 81 (54%) of parents or caregivers were females. The age of parents or caregivers ranges from 22 to 60 years, with mean (standard deviation of 36.91 (8.01) years. Regarding the employment status, 73 (48.7%) of them were employed. Majority of parents or caregivers were educated where 63 (42%) of them have attended college or university level education.

**Table 1:** Sociodemographic characteristics of parents or caregivers and paediatric patients underwent surgery in SPHMMC from June 1 to October 30, 2022

Characteristics	Categories	Freq. (%)
Patients gender	Male	107(71.3)
	Female	43(28.7)
Patients age	School age	53(35.3)
	Preschool age	97(64.7)
Parents or caregivers gender	Male	69(46.0)
	Female	81(54.0)
Caregivers educational Level	Not educated	8(5.3)
	Primary School	25(16.7)
	Secondary School	54(36.0)
	College/University	63(42.0)
Caregivers employment status	Not employed	77(51.3)
	Employed	73(48.7)
Patient age, Mean (SD)		6.4 (3.57) years
Patient weight, Mean (SD)		20.3 (7.59) kg
Parents or care givers age, Mean (SD) year		36.9 (8) years

### Clinical characteristics of participants

The clinical characteristics of paediatric surgical patients who were included in the study were presented in Table 2. Majority of study participants were classified under ASA-1 accounting 131 (87.3%). The result show that 79 (52.7%) of patients had difficulty separation compared to those who had good separation 71 (47.3%). This implies pediatric patients who had difficult separation were crying during separation from their parents or caregiver and require reassurance or premedication by anaesthesia provider. Further, 72(48%) of study participants were given premedication preoperatively before they were taken to the operation theatre. Result of the study also shows that 35(23.8%) of them had previous history of surgical procedure or anaesthesia exposure. Regarding surgery type, ENT and OMF surgeries accounts 60 (40%) followed by general paediatric surgery 57 (38%). The surgical procedures were done under general anaesthesia with tracheal intubation with ETT which accounts 103 (68.7%), 34 (22.7%) were done by LMA and 13 (8.6%) were done with facemask only.

**Table 2:** Clinical characteristics of paediatrics patients underwent surgery at SPHMMC from June 1 to October 30, 2022

Characteristics	Category	Freq. (%)
ASA classification	ASA 1	131(87.3)
	ASA 2	19(12.7)
Separation from parents or caregiver	Good separation	71(47.3)
	Difficult separation	79(52.7)
Premedication	No	78(52.0)
	Yes	72(48.0)
Previous surgical procedure or anesthesia	No	115(76.7)
	Yes	35(23.8)
Techniques of general anesthesia	GA/Facemask	13(8.7)
	GA/LMA	34(22.7)
	GA/ETT	103(68.7)
General anesthesia induction agent	Ketamine	5(3.3)
	Propofol	58(38.7)
	Ketofol	43(28.7)
	Thiopental	7(4.7)
	Halothane + Ketamine	8(5.3)
	Halothane + Propofol	16(10.7)
	Halothane + Ketofol	13(8.7)
Analgesia	Opoids	102(68.1)
	Non-opoids	15(10.0)
	Opioids + Non-Opioids	17(11.3)
	Caudal + Opioids	9(6.0)
	Caudal + Non-Opioids	7(4.7)
Extubating techniques	Deep extubation	28(18.7)
	Awake extubation	109(72.6)
Propofol administration at the end of procedure		
	No	109(72.7)
	Yes	41(27.3)
Types of surgery	General pediatric	57(38.0)
	ENT, OMF surgery	60(40.0)
	Ophthalmic surgery	17(11.3)
	Orthopedic and plastic	16(10.7)
Arrival status to PACU	Awake	97(64.7)
	Asleep	53(35.3)
Anesthesia duration	$<1$ hr	23(15.3)
	$\ge 1$ hr	127(84.7)
Duration of surgery	$<1$ hr	54(36.0)
	$\ge$ 1 hr	96(64.0)

Regarding an induction anaesthetic agent used for study participants intraoperatively, 58 (38.7%) were given propofol, and 43 (28.7%) of patients received Ketofol. Inhalational agent (halothane) was used in addition to intravenous induction agent for 37 (24.7%) patients who underwent surgical operation during study period. Further, 102 (68.1%) and 15(10.0%) of study participants took opioids and non-opioids as intraoperative analgesia, respectively. About 17 (11.3%) patients took combination of opioids and non-opioids analgesia, and only 9 (6.0%) and 7 (4.7%) took caudal analgesia with opioids and non-opiods, respectively (See Table 2). Furthermore, 41 (27.3%) of patients were provided propofol at the end of inhalational anaesthesia before extubation. Arrival conditions of the patients to PACU were also presented in Table 2 which shows that majority of patients were awake accounting 97 (64.7%). The mean (SD) of anaesthesia and surgery duration were 99.8 (58.14) and 82.8 (56.06) minutes, respectively. About eighty five and sixty four percent of all patients underwent anesthesia and surgery duration for over an hour, respectively (see Table 2).

### Magnitude of emergence agitation

**Fig. 1:**
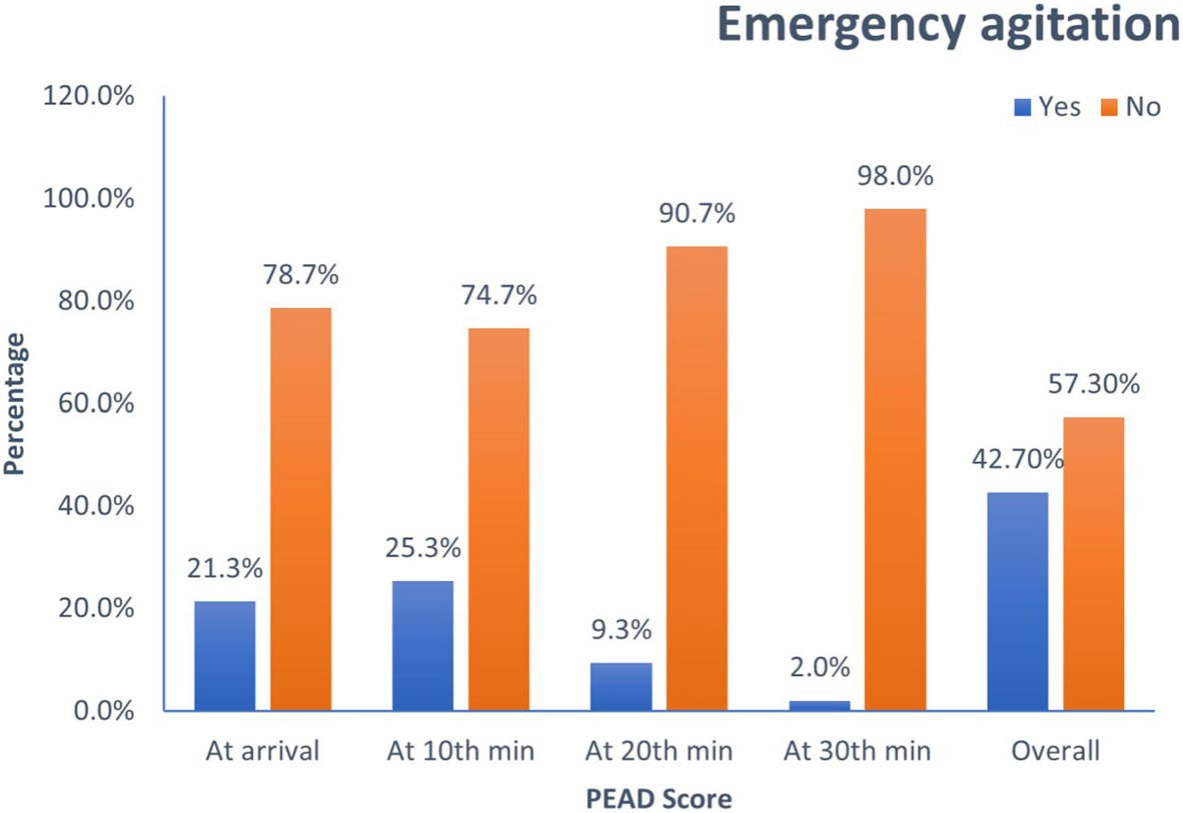
Percentage distribution of postoperative EA at follow up times of paediatric surgical patients in SPHMMC from June 1 to October 30, 2022

**Fig. 2:**
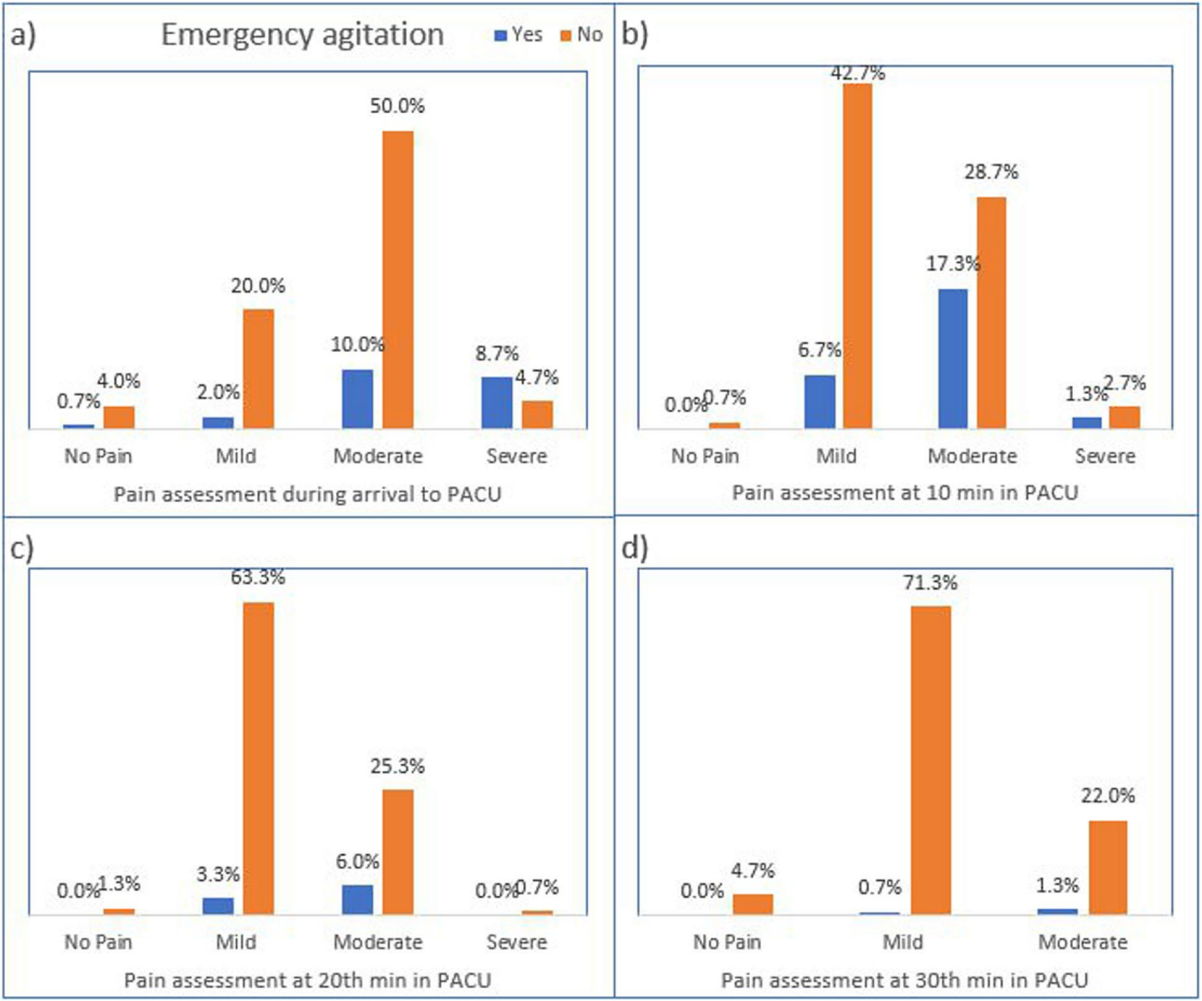
Postoperative emergence agitation by pain severity of paediatric patients who underwent surgery during follow up period at PACU in SPHMMC from June 1 to October 30, 2022

The overall magnitude of EA among the study participants during the study period was 42.7% as shown in Fig. 1. The mean ± SD of EA duration was 8.39 ± 4.45 minutes (Table 6). Furthermore, the magnitude of EA was compared at arrival, 10, 20 and 30 minutes of PACU stay where the magnitude was higher at 10th minutes (25.3%). The result also shows that the risk of developing EA after 20 minutes of PACU stay is decreased to 9.3% as shown in Fig. 2.

### Distribution of emergence agitation by socio-demographic characteristic

Distribution of emergence agitation among socio-demographic characteristics of pediatric surgical patients included in the study is presented on Table 3. It was observed that among male study participants 43% of them developed EA and among females, 42% of them developed EA. The magnitude of EA was high in preschool age patients where 49.5% of them developed EA. The majority of study participants were classified under ASA 1 which accounts 131(87.3%) among which 55(42%) developed EA. Among those pediatric surgical patients who has difficult separation, 43(54.4%) of them developed EA. About fourteen patients or forty percent of pediatric surgical patients exposure to previous surgical procedure or anesthesia have developed EA.

**Table 3:** Distribution of emergence agitation by socio-dempographic characteristics of parents or caregivers and paediatric patients who underwent surgery in SPHMMC from June 1 to October 30, 2022

		Emergence agitation developed		
Characteristics	Categories	Yes (n=64)	No (n=86)	
		Freq. (%)	Freq. (%)	$\chi ^2$(*p*-value)
Patients gender	Male	46 (43.0)	61(57.0)	0.016(0.899)
	Female	18(41.9)	25(58.1)	
Patients age	School age	16(30.2)	37(69.8)	5.217(0.022)
	Preschool age	48(49.5)	49(50.5)	
ASA classification	ASA 1	55(42.0)	76(58.0)	0.197(0.657)
	ASA 2	9(47.4)	10(52.6)	
Separation from parents or caregiver				
	Good separation	21(29.6)	50(70.1)	9.442(0.002)
	Difficult separation	43(54.4)	36(45.6)	
Previous surgical procedure or anesthesia				
	No	48(42.8)	67(57.2)	0.133(0.716)
	Yes	14(40.0)	21(60.0)	
Parents or caregivers gender	Male	27(39.1)	42(60.9)	0.653 (0.419)
	Female	37(45.7)	44(54.3)	
Caregivers educational level	Not educated	2(25.0)	6(75.0)	1.475(0.688)
	Primary School	12(48.0)	13(52.0)	
	Secondary School	22(40.7)	32(59.3)	
	College/University	28(44.4)	35(55.6)	
Caregivers employment	Not employed	35(45.5)	42(54.5)	0.503(0.478)
	Employed	29(39.7)	44(60.3)	

### Distribution of emergence agitation by anaesthesia and surgical variables

The distribution of EA by patient pre-medication was displayed in Table 4. Among 72(48%) patients who took premedication, 37(51.4) have developed EA while among the patients who did not take premedication, only 27(34.6%) developed EA. The magnitude of EA was observed high for patients who took premedication before surgery. Furthermore, there was statistically significant association between premedication and emergence agitation ($\chi ^2 = 4.306$, *p*-value = 0.038).

**Table 4:** Distribution of emergence agitation by anaesthesia and surgical characteristics of paediatric patients who underwent surgery in SPHMMC from June 1 to October 30, 2022

		Emergence agitation developed		
	Categories	Yes (n=64)	No (n=86)	$\chi ^2$
		Freq. (%)	Freq. (%)	(*p*-value)
**Anaesthesia-related characteristics**				
Premedication	No	27(34.6)	51(65.4)	4.306(0.038)
	Yes	37(51.4)	35(48.6)	
Techniques of general anesthesia	GA/Facemask	3(23.1)	10(76.9)	4.972(0.083)
	GA/LMA	11(32.4)	23(67.6)	
	GA/ETT	50(48.5)	53(51.5)	
General anesthesia induction agent				
	Ketamine	2(40.0)	3(60.0)	3.261(0.775)
	Propofol	24(41.4)	34(58.6)	
	Ketofol	22(51.2)	21(48.8)	
	Thiopental	3(42.9)	4(57.1)	
	Halothane + Ketamine	2(25.0)	6(75.0)	
	Halothane + Propofol	5(31.2)	11(68.8)	
	Halothane + Ketofol	6(46.2)	7(53.7)	
Analgesia	Opoids	44(43.1)	58(56.9)	0.507(0.973)
	Non-opoids	6(40.0)	9(60.0)	
	Opioids + Non-Opioids	8(47.1)	9(52.9)	
	Caudal + Opioids	3(33.3)	6(66.7)	
	Caudal + Non-Opioids	3(42.9)	4(57.1)	
Extubating techniques	Deep extubation	13(46.4)	15(53.6)	0.175(0.678)
	Awake extubation	42(42.0)	58(58.0)	
Propofol administration at the end of procedure				
	No	59(54.1)	50(45.9)	21.41(<0.001)
	Yes	5(12.2)	36(87.8)	
Arrival status to PACU	Awake	34(35.1)	63(64.9)	2.526(0.011)
	Asleep	30(56.6)	23(43.4)	
**Surgical-related characteristics**				
Types of surgery	General pediatric	20(35.1)	37(64.9)	6.305(0.098)
	ENT, OMF surgery	32(53.3)	28(46.7)	
	Ophthalmic surgery	8(47.1)	9(52.9)	
	Orthopedic and plastic	4(25.0)	12(75.0)	
Anesthesia duration	$<1$ hr	9(36.1)	14(60.9)	0.136(0.706)
	$\ge 1$ hr	55(43.3)	72(56.7)	
Duration of surgery	$<1$ hr	25(46.3)	29(53.7)	0.454(0.500)
	$\ge$ 1 hr	39(40.6)	57(59.4)	

It was observed that among 103 (68.7%) of paediatric surgical patients were done under GA/ETT technique, 50 (48.5%) have developed emergence agitation. Further, among 34 (22.7%) and 13 (8.6%) of paediatric surgical patients were done under general anaesthesia with GA/LMA and GA/facemask techniques, 11 (32.4%) and 3 (33.1%) have developed EA (see Table 4). However, the chi-square test association did not show association between between techniques of general anesthesia and emergence agitations ($\chi ^2$ = 4.972, *p*-value = 0.083).

**Table 5:** Univariable and multivariable logistic regression analysis of factors associated with EA among paediatric surgical patients in SPHMMC from June 1 to October 30, 2022

	Odd ratio estimates			
Covariates	COR, [95% CI]	*p*-value	AOR, [95% CI]	*p*-value
Age category				
School age	Reference			
Preschool age	2.265, [1.115, 4.602]	0.024	1.188, [0.445, 3.175]	0.731
Separation from parents or caregiver				
Good separation	Reference			
Difficult separation	2.844, [1.448, 5.586]	0.002	1.741, [0.723, 4.689]	0.201
Propofol administration at the end of procedure				
No	Reference			
Yes	0.118, [0.043, 0.323]	<0.001	0.104, [0.05, 0.305]	$<0.001$*
Types of surgery				
General pediatric	Reference			
ENT, OMF	2.114, [1.005, 4.448]	0.048	2.341, [1.051, 5.211]	0.037*
Ophthalmic	1.644, [0.549, 4.924]	0.374		
Orthopedic, plastic	0.616, [0.175, 2.164]	0.450		
Arrival status to PACU				
Asleep	Reference			
Awake	0.414, [0.209, 0.821]	0.012	0.456, [0.209, 0.904]	0.048*

*Statistically significant

Regarding the surgical related factors, ENT and OMF surgical procedures account the majority with 60 (40.0%) among which 32(53.3%) developed EA, 57 (38.0%) accounts for general paediatric surgeries among which 20 (35.1%) have developed EA. More than half of paediatric patients who have had ophthalmology surgery have developed EA, while one in four of paediatric patients who underwent orthopaedic and plastic surgery have developed EA. . Propofol administration at the end of inhalational anesthesia was assessed in this study and 41 (27.3%) of them were given propofol from which only 5(12.2%) of them developed EA (see Table 4). Furthermore, among study participants, 97 (64.7%) of them were taken to PACU awake where 34(35.1%) of them developed EA. The chi-square test of association result show that there was significant association between pediatric surgical patients who were taken to PACU and EA ($\chi ^2 = 2.526$, *p*-value = 0.011).

### Distribution of emergence agitation by pain assessment

The association of postoperative pain and emergence agitation were presented in Fig. 2 where both pain and emergence agitation were assessed at arrival, 10, 20, and 30th minutes of PACU admission. Accordingly, at arrival to PACU 90 (60%) of study participants had moderate pain among which 15(10%) of them developed emergence agitation. At 10th minutes of PACU stay, although most of the study participants had mild pain 49.4%, the risk of emergence agitation was higher among study participants who had moderate pain. In general, from Fig. 2 it was observed that those study participants who had moderate and severe pain were at increased risk to develop emergence agitation.

### Factors associated with emergence agitation

The significance of the associated factors was investigated by conducting marginal two by two cross tabulation using the Pearson chi-squared test statistics and the associated *p*-value. The result were reported in Tables 3 and 4 for the socio-demographic and clinical related characteristics, respectively. The result of a crude and adjusted odd ratio with the 95% confidence interval of factors associated with EA of pediatric surgical patients was reported in Table 5. The results show that preschool aged pediatric patients tend to show higher risk of emergence agitation development than school aged patients with (COR = 0.441, 95% CI: 0.217 - 0.897, p-value = 0.022). The chi-square test of association also supports this conclusion with a chi-square statistic of 5.217 and *p*-value 0.022. Similarly, difficult separation of patients from parents or caregivers before surgical procedures was associated with increased risk of EA (COR = 2.844, 95%: 1.448 - 5.586, and P-value =0.002). The chi-square test of association also supports this conclusion with a chi-square statistic of 9.442 and *p*-value 0.002. This show that difficult separation increases the risk of EA by three times when compared to pediatric patients who have good separation from their parents or caregivers. However, the characteristics such as, patients gender, ASA classification, previous surgical or anesthesia exposure were not associated with emergence agitation (see, Table 5). With reference to the general pediatric surgery type, patients with ENT and OMF surgery types were associated with the development of EA (COR = 2.114, 95% CI: 1.005 - 4.448, *p*-value = 0.048) while ophthalmology and orthopedic and plastic surgeries were not associated with EA. Further, the study showed that there was significant association between propofol administration at the end of inhalational anesthesia and EA compared to those who did not take propofol(COR = 0.118, 95% CI: 0.043 - 0.323, *p*-value < 0.001). Patients who were taken to PACU awake had lower risk of developing EA compared to those asleep(COR = 0.414, 95% CI: 0.029 - 0.821, *p*-value= 0.011) (see Table 5).

Multiple regression logistic analysis of this study showed propofol administration at the end of procedure significantly decreased the occurrence of EA (AOR = 0.104, 95% CI: 0.035 - 0305, *p*-value < 0.001). Similarly, the arrival of the patient to PACU awake significantly decreased the risk of EA compared to those patients who arrived at PACU asleep (AOR = 0.456, 95% CI: 0.209 - 0.994, *p*-value = 0.048). In addition, ENT and OMF surgeries significantly increased the risk of EA more than two times compared to other types of surgery (AOR = 2.341, 95% CI:1.051 - 5.211, *p*-value = 0.037). Generally, this study result shows that propofol administration at the end of procedure, patient arrival to PACU awake, ENT and OMF surgeries independently affect emergence agitation. Preschool age of patients and those who had difficult separation from parents did not have association on multivariate logistic regression (Table 5).

### Management of emergence agitation

Adverse events after emergence agitation were presented in Table 6 where the result shows that removal of iv cannula, drains, or catheters was the major adverse events observed among those who developed emergence agitation post operatively which accounts 28 (43.8%). Furthermore 16 (25%) of them had injury to themselves or caregivers. Regarding the management done for emergence agitation at PACU, 54 (84.3%) of study participants who developed EA were consoled by their parents or caregivers. Physical restraints and medication as a treatment were used in addition to family consolation in 15 (23.4%) and 20 (31.2%), respectively. The mean and SD of time stayed in recovery for those who developed EA was 123.02 ± 22.74 minutes compared to non-agitated patients which is 106.49 ± 17.71 minutes. This result shows that time in recovery stay was longer for those who developed EA (see Table 6).

**Table 6:** Management related findings of emergence agitation in paediatric surgical patients underwent surgery at SPHMMC from June 1 to October 30, 2022

Variables	Frequency (N)	Percentage (%)
Adverse events after emergence agitation		
Injury to themselves or Caregivers	16	25
Falling accident	2	3.2
Removal of iv cannula, drains or Catheters	28	43.8
Interventions done		
No interventions done	11	17.1
Consoled by family	54	84.3
Consoled by family + Physical restraints	15	23.4
Consoled by family + Medication	20 3	1.2
EA duration (mean ± SD)	8.39 ± 4.45 minutes	
Time stayed in PACU in agitated patients (mean ± SD)	123.02 ± 22.74 minutes	
Time stayed in PACU for non-agitated (mean ± SD)	106.49 ± 17.71 minutes	

## Discussion

In this cross-sectional prospective study, the magnitude of emergence agitation, its management and associated factors were assessed among 150 paediatric surgical patients 2 - 14 years old who underwent surgery under general anaesthesia in SPHMMC, Addis Ababa, Ethiopia.

According to the current study, the magnitude of EA was 42.7% which is a higher percentage even though it is difficult to compare with other studies due to variations in protocols and in the definition of EA. This result was in the range of reports from international literature which showed the magnitude of EA ranges between 10% to 80% [2, 3]. The magnitude of EA in the current study was comparable to study done in Thailand [10] which showed an incidence rate of 43.2%. Studies done in Gonder university and Addis Ababa university shows that the incidence of EA was 52.3% and 49% respectively [8, 9] which is a higher rate than the current study. The reason for this variability might be due to different scoring methods used for assessment of EA which might have inter-observers’ variability.

The present study showed that preschool children (< 7years old) were highly likely to have EA compared to school age groups (COR=2.265, 95% CI: 1.115 - 4.602, *p*-value = 0.024). This study result was in accordance with research done by Eshete et al. [8], Dahman et al. [10], and Choi et al. [16]. The reason behind this is preschool children may be psychologically less mature and less able to cope up awakening from anaesthesia.

The association between difficult separation from families or caregivers before surgery and EA has been reported in the previous literatures [9, 11, 17]. The current study shows that difficult separation of patients from parents or caregivers had three times more risk to develop EA than those who have good separation even though multivariate analysis did not find an association. This might be due to small sample size in the current study and subjective nature of the assessment of parental separation behaviour.

The current study result shows that paediatric surgical patients who received propofol at the end of inhalational anaesthesia had a lower risk of EA (AOR = 0.104, 95% CI: 0.035 - 0.305, *p*-value < 0.001). This result was in line with a systematic review and meta-analysis done in 2015 which showed prophylactic propofol (1 mg/kg) given at the end of inhalational anaesthesia appears to be effective for reducing the incidence and severity of EA in children emerging from general anaesthesia [18]. Another research done in Gondar also found that the risk of EA was significantly decreased in those patients who were given propofol [8] which support the current study finding. The reason behind this may be due to propofol leading to smooth recovery from anaesthesia.

The choice of general anesthesia induction agent was not significantly associated with emergence agitation. It was observed that most of patients who received anesthesia induction did not experience emergence agitation. In fact, although ketamine can contribute to sedation and analgesia while keeping cardiovascular stability [19], and providing bronchodilation [20], it has been shown to influence the incidence of EA [21]. However, due to the limited number of patients were administered ketamine as a induction agent, this study did not investigate its effects on the development of emergence agitation. Furthermore, small number of patients were administered other induction agents or combination of other induction agents, making it difficult to examine the effects on the development of emergence agitation.

Regarding the surgical types, this study showed that ENT and OMF surgeries were more than two times more associated to EA than other types of surgeries (AOR = 2.341, 95% CI: 1.051 - 5.211, *p*-value =0.037). This finding was in support of studies [22, 23], where otorhinolaryngologic procedures and tonsillectomy surgery were independent predictors of EA. The reason for this increased risk might be due to a feeling of suffocation during emergence from anaesthesia as these kinds of procedures are done in the oral cavity or near the oral cavity. This study result showed that paediatric surgical patients who had moderate to severe pain during the follow-up period were more likely to develop EA than those with mild or no pain. Randomised double blind study done by Sethi concluded that children with higher pain scores were highly likely to have EA [24]. In contrast, a study done by Eshete [8] found no association between pain and emergence agitation. This difference may be due to reports of higher emergence agitation in children even though they underwent painless procedures. In addition, pain assessment tools used may have an inter-observer’s difference.

Multivariate analysis in the current study showed that there was a significant association between arrival of patients to PACU awake and EA when compared to those asleep (AOR = 0.456, 95% CI: 0.209 - 0.994, *p*-value = 0.048). Hence, those patients brought to PACU awake after extubation had a lower risk of emergence agitation than those brought asleep. In contrast to this study, a prospective descriptive study done in South Africa found that there was no significant difference in EA among patients brought to PACU extubated awake and extubated asleep [25]. This difference may be in our study all patients were brought to PACU extubated in contrast the majority of the patients were brought to PACU intubated in the study done in South Africa. Additionally, awake children are more aware of their surroundings especially their parents, which decreases the risk of emergence agitation in the presence of their parents [18]. This study also assessed interventions done for those patients who developed emergence agitation at PACU. Accordingly, the majority of the patients received non pharmacological treatment like consolation by their parents or caregivers (83.3%) by allowing the parents to be with their child during their recovery stay. In addition to this, medications were additionally used in only 31.2% of patients. The result of this study was in line with a study done in South Africa [25], where non-pharmacological strategies were advocated as a reasonable treatment method. Furthermore, this study depicted that initial interventions should be tried with non-pharmacologic treatment and medications should be reserved if EA is not improved and if pain is associated with EA.

In this study time of recovery stay was prolonged among surgical pediatrics patients who developed EA during study period with mean (SD) of 123.02 ± 22.74 minutes similar to a study done in Jamaica by Gooden [11]. The prolonged recovery time most likely resulted from the need of pharmacologic treatment and other supportive treatments necessary to manage EA.

Since emergence agitation was measured using the PAED score measurement tool even though it is the only validated and reliable [13, 14], this study is limited to the subjective nature of the response and may result in inter-observers’ variability. In addition, the small sample size used in this study may limit its generalizability to the study populations.

## Conclusion

In this study the magnitude of EA is high which needs anaesthesiologist to take measures to minimize the occurrence or shorten the duration of EA. According to this study finding, propofol administration at the end of inhalational anaesthesia and arrival of the patient to PACU awake significantly decreased development of EA. Even though there is no association on multivariate analysis, patients in preschool age and having difficult parental separation have higher rates of development of EA. ENT and OMF surgeries were also significantly associated with EA which needs precaution to minimize the magnitude. Furthermore, those paediatric surgical patients who had moderate and severe pain were associated with EA. Time stayed in recovery were also prolonged in those patients who developed EA.

## Data Availability

The datasets used and/or analysed during the current study are available from the corresponding author on reasonable request.

## Abbreviations

AOR: Adjusted Odd Ratio
ASA: American society of Anaesthesiology
COR: Crude Odd Ratio
EA: Emergence Agitation
ED: Emergence Delirium
ENT: Ear, Nose, Throat
FLACC: Face, leg, activity, cry, consolability
GA: General Anaesthesia
KG: Kilogram
OMF: Oral and maxillofacial
PACU: Post-anaesthetic Care Unit
PAEDS: Paediatric Anaesthesia Emergence Delirium Score
PICU: Paediatric intensive care unit
PMB: Postoperative Maladaptive Behaviour
SPHMMC: Saint Paul’s Hospital Millennium Medical College
SPSS: Statistical Package for The Social Sciences
